# Polysaccharide immunization and colorectal cancer: A systematic review and network meta-analysis

**DOI:** 10.3389/fnut.2022.961507

**Published:** 2022-07-22

**Authors:** Yuefeng Chen, Xinnan Pan, Baoming Tian, Yajun Hu

**Affiliations:** ^1^Department of Medical College of Shaoxing University, Shaoxing, China; ^2^Department of Medical College of Wenzhou Medical University, Wenzhou, China; ^3^College of Food Science and Technology, Zhejiang University of Technology, Hangzhou, China; ^4^Zhejiang Institute of Modern TCM and Natural Medicine Co., Ltd., Hangzhou, China; ^5^School of Pharmaceutical Sciences, Zhejiang Chinese Medical University, Hangzhou, China

**Keywords:** colorectal cancer, CD24, prognosis, polysaccharides, immune

## Abstract

Polysaccharides have a variety of biological activities, and in the anti-tumor field, they produce tumor suppressive effects by regulating the polarization of tumor-associated macrophages (TAMs). In immunotherapy, it has significant activities in modulating cytokines and antibody production. We reviewed them and selected CD24, an immune target, for meta-analysis with colorectal cancer (CRC) to investigate the correlation between CD24 expression and CRC. Correlation of CD24 positive expression with clinical-pathological features: age, sex, Duke’s stage, diameter, depth of invasion, degree of differentiation, and lymph node metastasis. It showed that: CD24 expression in CRC was significantly correlated with advanced nuclear grade of CRC, lymph node metastasis, Duke’s stage of CRC and age of CRC patients, while there was no significant correlation with gender, tumor diameter and invasion depth. The aim is to clarify the specific mechanism of polysaccharide immune anti-tumor, combined with targeted site-specific anti-solid tumor.

TCM (Traditional Chinese medicine) has multiple benefits by altering the microenvironment and enhancing the action of the immune system (1). Plant polysaccharides as TCM-derived components have been widely studied. Many biological properties of plant polysaccharides have been discovered, including anticancer, antioxidant, and immunomodulatory effects (2). Ginseng polysaccharide is effective against non-small cell lung cancer (3, 4), astragalus polysaccharide is effective against liver cancer (5), and shiitake mushroom polysaccharide is effective against CRC (6). Jujube polysaccharides also improve intestinal flora and inhibit CRC progression (7). TAMs have been implicated in anti-tumor processes. M1 macrophages can limit tumor cell proliferation in diverse tumor microenvironments (TME), but M2 macrophages have pro-tumor effects. TAMs infiltration and polarization in TME can be regulated by plant polysaccharides, which can enhance M1 polarization, M2 to M1 polarization, and inhibit M2 function. *Radix Codonopsis* polysaccharide increases macrophage activation and polarization to M1, impairs TME, and prevents cancer cells from evading the immune system (8). Epimedium polysaccharide modulates the TME, reverses TAM phenotype, boosts T cell expression, and boosts tumor immunity (9). The CD14 pathway is activated by Poria polysaccharide, which promotes the conversion of M2 to M1. Plant polysaccharides mainly regulate TAMs in terms of regulating infiltration and regulating polarization, which play a role in improving the tumor microenvironment and anti-tumor effects. The study of signaling pathways plays a crucial role in this job. In TAMs, IAPS-2 (An acidic polysaccharide extracted from the roots of an herb called llex asprella) inhibits STAT3 phosphorylation while increasing STAT1 phosphorylation, facilitating M1 polarization (10). Ganoderma lucidum polysaccharide PSG-1 induces activation of TAMs through TLR4-mediated NF-κB and MAPK signaling pathways, increases TNF-α and NO expression, promotes M1 properties, and exerts anti-tumor activity (11). In addition, dendrobium officinale polysaccharide can significantly inhibit tumor growth in mice by directly targeting TLR2 and promoting the polarization of TAMs toward M1 (12). JAK/STAT, PI3K/Akt, JNK, and other signaling pathways are also involved in modulating TAMs phenotype.

NF-κB pathway, PD-L1 and leukocyte differentiation antigens are important targets for the biological activities of proliferation, differentiation, and migration of solid tumors such as colorectal. EG, MG, HXRARG, and HXRA, four polysaccharide active components of ryegrass, induce P65 and IκBα phosphorylation to begin the NF-κB pathway, increase NO, TNF-α, and IL-6 production, and boost immunological activity (13). *Ganoderma lucidum* polysaccharide inhibits STAT3 phosphorylation, suppresses PD-L1 expression, boosts PD-L1 antibody effectiveness, and reduces the side effects of targeted inhibitors such wasting and anemia (14). The expression of CD40, CD53, CD80, CD86, and CD83 molecules on the surface of dendritic cells was significantly increased by Erysipelas polysaccharides, as was the expression of cytokines such as IL-12 p40 and IL-10 (15, 16). Momordica charantia was able to reduce the expression levels of inflammatory factors, such as IL-1β, IL-6, IFN-γ, and TNF-α (17).

CD24, as the major histocompatibility complex on the surface of dendritic cells, is a highly glycosylated mucin-like cell surface adhesion molecule with a relative molecular weight of 35–45 kDa. It is anchored to the cell surface by Glycosylphosphatidylinositol (GPI). It regulates cell adhesion in cell-cell cell-matrix interactions and is significantly related to tumor cell infiltration and proliferation (18, 19). Under physiological conditions, it is expressed in developmental or regenerated tissues and granulocytes, pre-B cells, keratinocytes, and renal tubular epithelial cells (20–22). Barkal et al. found on tumor cells that CD24 was able to bind to the protein Siglec-10 on macrophages and inhibit the anti-tumor effect of phagocytosis. And the CD24^–^ deficient tumors infiltrating TAMs showed enhanced phagocytosis and lower growth than controls. After cycle culture, polyclonal CD24^–^ tumors became mostly CD24^+^, consistent with the selection of CD24^–^ cells by TAMs and the appearance of CD24^+^ cell subclones without the double allele CD24 deletion. Suggesting that TAMs-mediated increase in CD24^–^ cell clearance is responsible for the reduction in tumor load (23). Not surprisingly, there are few studies that address such issues as polysaccharide regulation of CD24 or CD24 affecting polysaccharide regulation. Extensive data search was able to identify that they could have a common role in TAMs polarization, etc. In summary, we can block CD24 expression by monoclonal antibodies to enhance the phagocytosis of polysaccharide-regulated TAMs and significantly improve the anti-solid tumor effect.

CRC is a long-term, multi-programmed process involving in a series of oncogenes, tumor suppressor genes, and RNA gene mutations, resulting in changes in the function of important intracellular regulatory factors (24). Several studies have shown that high expression of CD24 in tumor cells and its involvement in CRC cell proliferation and invasion is a poor prognostic factor for CRC (25). Colorectal adenoma, a precancerous lesion of CRC, has an expression of CD24 comparable to that found in CRC at 90%. In contrast, CD24 expression in normal tissue near the lesion was almost 0 (26). Therefore, a complete understanding of the relationship between the expression of CD24 in CRC and its clinical characteristics is of great significance for the treatment and prognosis of CRC.

## Correlation between CD24 and colorectal cancer

It is well known that colorectal adenoma is the most crucial precancerous disease of CRC. Studies have shown that the positive rate of CD24 is as high as 60% when the diameter of colorectal polyp is less than 2.0 cm. Moreover, CD24 increased with age, the diameter of colorectal polyps, the type of dysplasia of colorectal polyps, the metastasis of CRC, and the degree of differentiation of CRC, which showed a significant positive correlation (27, 28). This suggests that the change in the expression of CD24 is an early event in the development of CRC. At the same time, a series of experiments indicate that CD24 is involved in the occurrence and development of CRC (29–31). For example, Tan et al. used flow cytometry to study the expression of CD24 on peripheral blood CD3 cells in patients with CRC (observation group) and healthy controls (control group). It was found that the fluorescence intensity of CD24 on CD3 cells in the observation group was 337.02 ± 27.92 and that in the control group was 293.84 ± 9.54. There was a significant difference between the two groups. Some studies have shown that CD24 has a great relationship with CRC treatment and can be used as a target for targeted therapy of CRC (32, 33).

## The role of CD24 in the growth, invasion, recurrence, and metastasis of colorectal cancer

CD24 is highly expressed in most tumor specimens and is significantly related to the infiltration and proliferation of tumor cells. CD24 is a glycosylated phosphatidylinositol cell surface protein that mediates cancer cells, activated platelets, vascular endothelial cells, lymphatic circulation, and blood migration, resulting in tumor invasion and distal metastasis (34, 35). However, there is no transmembrane structure in CD24. When CD24 is expressed in CRC cell lines, some literature has shown that CD24 transduces the MAPK signal pathway through the activation of Src family kinases to promote the growth and invasion of CRC and enhance the invasive ability of CRC cells through Lyn. Lyn is one of the members of the Src family kinase family.

It has been reported that the expression of CD24, CD24 in 92.5% of human CRC tissue, can promote the growth of CRC cells. The positive expression rate of CD24 in the tumor diameter ≥ 5 cm group is as high as 87.0%, which is significantly higher than that in the tumor diameter < 5 cm group. To some extent, this shows that CD24 promotes the growth of CRC cells.

The expression of CD24 in liver metastasis of CRC increases gradually, and a significant positive correlation can indicate the metastasis of CRC. P-selectin is a calcium-dependent endogenous lectin, rapidly expressed on the surface of activated vascular endothelial cells and platelets induced by inflammation and trauma. P-selectin is the earliest recognized and the only known ligand of CD24. In the pathological state of CD24, the organ can capture tumor cells and form distant metastasis by binding to P-selectin on the surface of vascular endothelial cells of distant organs or by interacting with platelets (23, 36).

## Correlation between CD24 expression and clinicopathological features in colorectal cancer

CD24 gradually increased with the degree of differentiation of CRC, showing a significant positive correlation. With the increase of the degree of differentiation of CRC, the expression of CD24 gradually increased (37). The increase in the expression rate of CD24 can be used as the basis for the malignant transformation of colorectal polyps. With the increase in the diameter of colorectal polyps, the degree of polyp dysplasia, and the degree of differentiation of CRC, the expression rate of CD24 increases.

The expression level of CD24 in CRC is related to the clinicopathological features of CRC. The expression level of CD24 in CRC without serosa invasion was 0.7495 ± 0.0392, and in serosa invasion, CRC was 0.8935 ± 0.0521. The difference was statistically significant; that is, the expression level of CD24 in CRC increased with the degree of invasion of CRC.

## The correlation between the expression of CD24 in colorectal cancer and the prognosis of colorectal cancer

A colorectal adenomatous polyp is a crucial precancerous lesion of CRC. Early detection of colorectal adenomatous polyp is helpful to the prognosis of CRC, and it has been reported that 95% of CRC is malignant from adenoma (38). In the early stage of the multistage progression of CRC, CD24 is highly expressed and malignant (39). The expression rate of CD24 in normal mucosa, inflammatory polyp, tubular adenoma, villous adenoma, and adenocarcinoma of CRC increased gradually, and the expression rate of CD24 increased with the degree of differentiation of CRC. Therefore, early analysis of CD24 expression can reduce the probability of colorectal polyps deteriorating into CRC and promote the prognosis of CRC (37).

Su et al. showed that the average survival time of the CD24 low expression group was 55.299 months, while the average survival time of the high expression group was 36.324 months. There was a significant difference in survival rate between the low expression group and the increased expression group. The survival rate of the expanded expression group was lower than that of the joint expression group, which could be used as an independent factor affecting the prognosis of patients with CRC.

## Objectives

A meta-analysis of the correlation between CD24 expression and clinicopathological features and prognosis in colorectal cancer was conducted.

## Materials and methods

### Inclusion criteria

(1) The subjects were all people with CRC confirmed by pathology.

(2) It is necessary to include data on the correlation between the expression of CD24 in CRC and clinicopathological features.

### Exclusion criteria

(1) Editorials, reviews, previews, abstracts, letters, and non-human basic research.

(2) Research in which data cannot be extracted.

(3) The language of the literature except for Chinese and English.

(4) Research that does not contain up-to-date data has been repeatedly published.

### Retrieval strategy

The computer searches the Embase, PubMed, Web of Science. Subject search words include “CRC,” “CD24,” and so on. Free words include “age,” “sex,” “stage,” “diameter,” “depth of invasion,” “degree of differentiation,” “lymph node metastasis” and so on. The method of combination of subject words and free words is used for retrieval. The retrieval time is from establishing the database to April 1, 2022, to improve the literature search and accuracy. The list of references that have been included in the study is also searched manually to ensure the recall rate. In addition, manual search excludes important meetings in case reports, abstracts, reviews, letters, animal experiments, and oncology.

### Statistical method

#### Statistical software and analysis

All the data included in this meta-analysis were merged and analyzed by Review Manager 5.4 software. The *I*^2^-value judges the heterogeneity among the studies. If *I*^2^ > 50%, it can be considered that there is significant heterogeneity among the included studies, and the random effect model is selected to combine the effects; if *I*^2^ < 50%, it means that the included studies are homogeneous, and the fixed effect model is chosen to combine the results. Higgins et al. believe that the value of *I*^2^ is between 0 and 100%, and the higher the value of *I*^2^, the more significant the heterogeneity among the included studies. When the *I*^2^-value is 25%, it indicates mild heterogeneity among the included studies, and the existence of *I*^2^ = 50% suggests that there is moderate heterogeneity among the studies. In contrast, when *I*^2^ = 75%, it indicates a high degree of heterogeneity among the studies. It is generally believed that when *I*^2^ > 50%, the heterogeneity among the included studies is higher.

#### Sensitivity analysis

The heterogeneity between studies was detected by observing the value of *I*^2^. If there is moderate or high heterogeneity between studies, the source of heterogeneity needs to be determined by sensitivity analysis. After the single study was excluded, the effects of the remaining studies were excluded, and the heterogeneity was compared with the total heterogeneity. Suppose the combined heterogeneity changes significantly compared with the total heterogeneity or outside the 95% confidence interval of the point estimated total effect of the combined effect amount in the forest map of sensitivity analysis. In that case, the results of this study are unstable. The study should be analyzed in-depth to determine the source of heterogeneity to draw cautious conclusions.

#### Publication bias

There are few studies on the survival outcomes of patients in this meta-analysis, so this study uses Egger’s test and Begg’s test to evaluate whether the included studies had publication bias when the *P* > 0.10 of the Egger’s test and *P* > 0.05 of the Beggles test suggested no publication bias.

## Results

### Literature retrieval results

**FIGURE 1:**
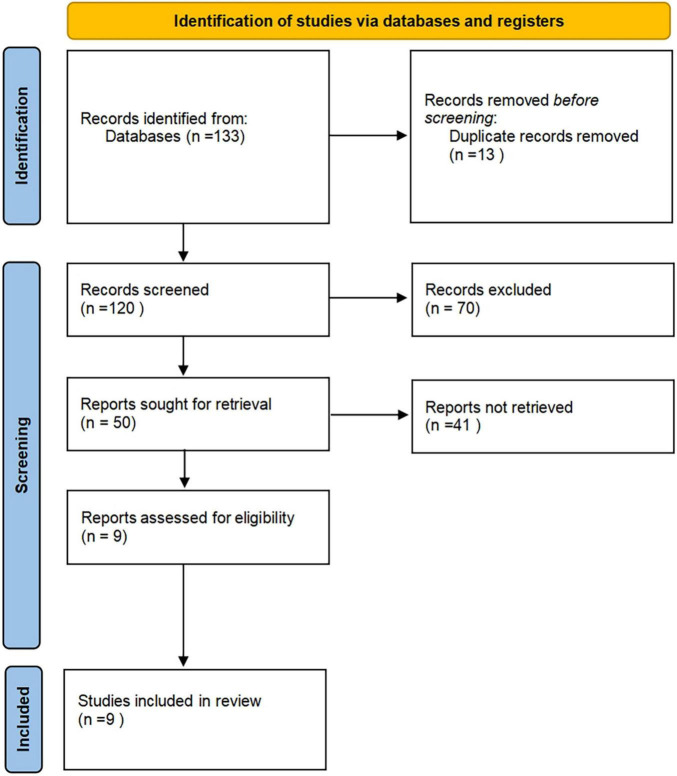
Document retrieval flow chart.

According to the established retrieval strategy, 133 related articles were retrieved electronically, and 13 duplicate articles were excluded. In strict accordance with the criteria of nano-arrangement, 70 pieces that were not following the meeting minutes, abstracts, reviews, non-human studies, and non-Chinese and English literature were excluded, and 50 other articles were excluded. After carefully reading the complete text, 41 articles that did not conduct a separate study on CD24 and whose data could not be extracted were banned. Finally, 9 studies that met the inclusion criteria were obtained, with 1,105 patients with CRC (Figure 1). [(1) Chen (40), Study of c-myc and CD24 expression in colorectal cancer and polyps. (2) Chen et al. (41), Expression of CD24 in colorectal cancer and its significance. (3) Su et al. (42), Expression of CD24 and Src in colorectal cancer tissues and their significance. (4) Su (43), The role and mechanism of Lyn in the regulation of colorectal cancer invasion by CD24. (5) Hua et al. (44), Expression of CD44 and CD24 in colorectal cancer and adenoma and their tumorigenic and invasive abilities. (6) Su (45), Expression of CD24 and CD166/ALCAM in colorectal cancer and their relationship with tumor cell proliferation and angiogenesis. (7) Shu (46), Study of CD24 and Lgr5 expression in colorectal polyps and colorectal cancer. (8) Xue et al. (47), Expression of CD24 and Lgr5 in colorectal polyps and colorectal cancer. (9) Zhang (48), Expression analysis of CD24 and Lgr5 in colorectal polyps and CRC].

### Correlation between CD24 expression and clinicopathological features in colorectal cancer

#### Correlation between the expression of CD24 in colorectal cancer and high-grade nuclear grade

**FIGURE 2:**
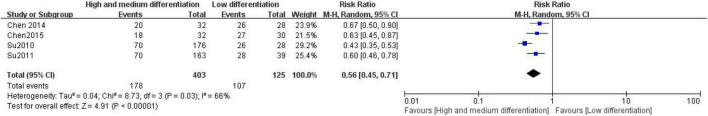
Forest map of correlation between CD24 expression and high-level nuclear classification.

A total of 4 studies were included in the evaluation of the correlation between the expression of CD24 and the degree of differentiation in CRC. A total of 528 patients with CRC were included, including 403 patients in the high and middle differentiation group and 125 patients in the low differentiation group (Figure 2). The heterogeneity test (*I*^2^ = 66%, *P* = 0.03) showed that the heterogeneity was small, so the fixed effect model combined the effects. The results show a significant correlation between the expression of CD24 in CRC and the degree of differentiation of patients (RR = 0.56, 95% CI:0.45–0.71, *P* < 0.00001).

#### Correlation between expression of CD24 and lymph node metastasis in colorectal cancer

**FIGURE 3:**
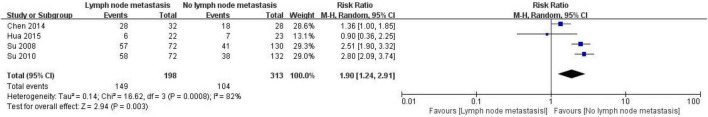
Forest map of correlation between CD24 expression and lymph node metastasis.

A total of 511 CRC patients were included in four studies to evaluate the correlation between CD24 expression and lymph node metastasis, including 198 patients with lymph node metastasis and 313 patients without lymph node metastasis (Figure 3). The heterogeneity test results showed that *I*^2^ was 82%, *P* = 0.0008. The results showed considerable heterogeneity among studies, so the random effect model combined the effect. The results showed a significant correlation between the expression of CD24 in CRC and lymph node metastasis (RR = 1.90, 95% CI: 1.24–2.91, *P* = 0.003).

#### Correlation between expression of CD24 in colorectal cancer and dukes staging

**FIGURE 4:**
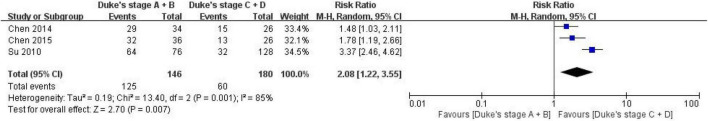
Forest map related to CD24 expression and dukes staging.

Three studies were included in evaluating the correlation between the expression of CD24 in CRC and Duke’s stage, including 125 patients with Duke’s stage A&B and 60 patients with Duke’s stage C&D stage (Figure 4). The heterogeneity test results showed that *I*^2^ = 85%, *P* = 0.001, suggesting that the heterogeneity among the studies was enormous, so the random effect model was used to combine the effect. The results showed a significant correlation between the expression of CD24 in CRC and Duke’s staging (RR = 2.08, 95% CI: 1.22–3.55, *P* = 0.007).

#### Correlation between age and expression of CD24 in colorectal cancer

**FIGURE 5:**
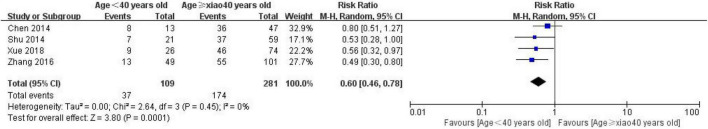
Forest map related to CD24 expression and age.

A total of 4 studies were included in the evaluation of the correlation between the expression of CD24 and age in CRC, including 281 patients in the age ≥ 40 group and 109 patients in the age < 40 groups (Figure 5). The heterogeneity test results showed that *I*^2^ = 0%, *P* = 0.0001, suggesting that the heterogeneity among the studies was small, so the random effect model was used to combine the effects. The results show a significant correlation between the expression of CD24 in CRC and the age of the patients (RR = 0.60, 95% CI: 0.46–0.7, *P* = 0.0001).

#### Correlation between gender and expression of CD24 in colorectal cancer

**FIGURE 6:**
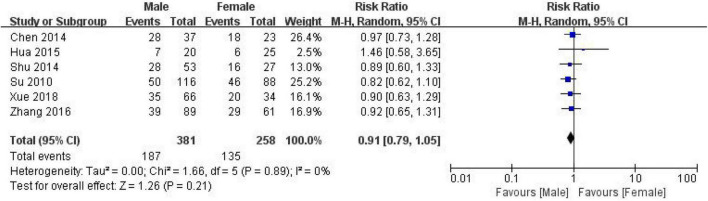
Forest map related to CD24 expression and gender.

639 CRC patients, including 381 males and 258 females, were included in 6 studies to evaluate the correlation between CD24 expression and lymph node metastasis in CRC (Figure 6). The heterogeneity results (*I*^2^ = 0%, *P* = 0.89) suggested that the heterogeneity among the studies was not significant, and the random effect model was selected to combine the effects. The results showed no significant correlation between the expression of CD24 and gender in CRC (RR = 0.91, 95% CI:0.79–1.05, *P* = 0.21).

#### Correlation between CD24 expression and tumor diameter in colorectal cancer

**FIGURE 7:**
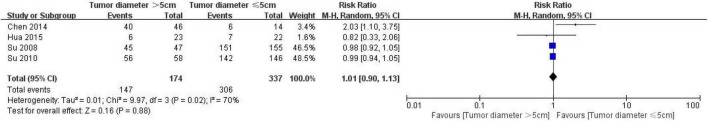
Forest map related to CD24 expression and tumor diameter.

A total of 511 patients with colon cancer were included in 4 studies to evaluate the correlation between the expression of CD24 in colon cancer and tumor diameter, including 174 patients with tumor diameter > 5 cm and 337 patients with tumor diameter ≤ 5 cm (Figure 7). The heterogeneity test results (*I*^2^ = 70%, *P* = 0.02) showed that the heterogeneity of each study was significant, so the random effect model was used to combine the effect. The results showed no significant correlation between the expression of CD24 in CRC and the diameter of the tumor (RR = 1.01, 95% CI: 0.90–1.13, *P* = 0.88).

#### Correlation between the expression of CD24 and the depth of invasion in colorectal cancer

**FIGURE 8:**
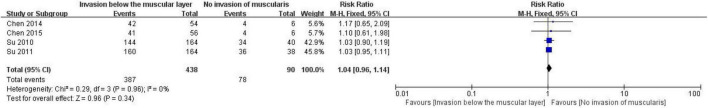
Forest map related to CD24 expression and infiltration depth.

A total of 528 patients with CRC were included in four studies to evaluate the correlation between CD24 expression and lymph node metastasis, including 387 patients with invasion of the muscular layer and 78 patients without charge of the muscular layer (Figure 8). The heterogeneity test results (*I*^2^ = 0%, *P* = 0.96) showed that the heterogeneity of each study was small, so the random effect model was used to combine the effect. The results showed no significant correlation between the expression of CD24 and the depth of invasion in CRC (RR = 1.04, 95% CI: 0.96–1.14, *P* = 0.34).

## Conclusion

The expression of CD24 in CRC was significantly correlated with a high-grade nuclear grade, lymph node metastasis, Duke’s stage, and age, but not with gender, tumor diameter, and depth of invasion.

## Discussion

Although the theoretical basis for plant polysaccharides’ immune antitumor and clinical applications has been established by their modulatory effects on TAMs, the current body of research is still limited to the bioactive effects on solid tumors, and the mechanism by which they cause this effect needs further investigation. There are several signaling pathways and targets associated with malignant tumors, thus it is yet unknown if there are regulatory mechanisms particular to polysaccharides that can prevent tumors or malignant disorders. TAMs are one of the possible targets of immunological anti-tumor since they are a significant part of TME. Since CD24 is related to the plant polysaccharide-regulating CRC pathway and has a regulatory polarizing impact on TAMs during the conclusion process, we chose CD24 for the CRC correlation study.

CD24 still faces significant challenges before it is used in the clinic. First of all, the clinical detection method of CD24 is immunohistochemical, and there is no unified standard for the definition of its critical value, which will be a significant limitation for the practical application of CD24 in clinical practice. Secondly, CRC with different differentiation may show other clinical characteristics and biological behavior. Still, due to the lack of data collected, our meta-analysis does not evaluate the relationship between CD24 and clinicopathological parameters of CRC with different differentiation, which needs further study.

In addition, there are some shortcomings in this meta-analysis, which are shown in the following aspects: (1) the methods for detecting tumor markers in our studies are all realized by immunohistochemistry. However, there is a certain degree of bias due to the differences in antibody types, drug concentrations, and critical values used in different studies. (2) The literature languages included in this study are limited to Chinese and English, excluding studies in other languages, which may lead to the exclusion of other appropriate studies, which may also lead to bias; (3) there are not many studies on the relationship between CD24 expression and prognosis, which is the reason why the results may be biased. (4) Most published studies lack the necessary data on patient treatment, creating additional inconsistencies and leading to potential selection bias.

Although this study has some limitations, this meta-analysis is still the first independent systematic evaluation of the correlation between CD24 expression and clinicopathology and prognosis in CRC. The meta-analysis of the above results shows that the expression of CD24 in CRC can reduce the overall and disease-free survival of patients with CRC and has a specific correlation with clinicopathological parameters, such as lymph node metastasis TNM stage and so on. It may have a particular reference value in future tumor differential diagnosis and targeted therapy. Examples include exosomal MiR-155 and MiR-665 as new hepatocellular carcinoma biomarkers (49), and ITGs as indicators of immune escape in NSCLC (50). Tumor-related markers can be analyzed from multiple perspectives and in multiple dimensions to consider the development of tumors and their prognosis. What is worth further thinking about is whether we can block the expression of CD24 in CRC in some way to inhibit the proliferation of tumor cells and prevent lymph node metastasis, to provide new ideas for clinical diagnosis and treatment to improve the prognosis of patients with CRC. However, the number of studies and sample size included in this meta-analysis is relatively tiny. In future studies, a large sample of prospective studies is needed to evaluate further the correlation between CD24 and clinicopathological features and prognosis to provide a sufficient theoretical basis for clinical treatment guidance of CD24.

Plant polysaccharides offer a significant advantage in the production of immune adjuvants due to their extensive diversity and low toxicity. Different immune adjuvants now in use play a vital role in tumor immunotherapy. Clinical application of several plant polysaccharides as effective all-natural immune adjuvants and anticancer adjuvants has previously occurred. In order to facilitate the targeted binding of CD24 antibodies and particular anti-CRC, they can also be molecularly altered.

## Author contributions

YC, XP, and BT: conception and design. YC and YH: administrative support, data analysis, interpretation, and independent evaluation of each eligible study. YC and XP: database management and statistical analysis, collection, and assembly of data. All authors contributed to the article and approved the final mauscript.

## Conflict of interest

BT was employed by the company Zhejiang Institute of Modern TCM and Natural Medicine Co., Ltd. The remaining authors declare that the research was conducted in the absence of any commercial or financial relationships that could be construed as a potential conflict of interest.

## Publisher’s note

All claims expressed in this article are solely those of the authors and do not necessarily represent those of their affiliated organizations, or those of the publisher, the editors and the reviewers. Any product that may be evaluated in this article, or claim that may be made by its manufacturer, is not guaranteed or endorsed by the publisher.
